# Pathways to psychiatric care for mental disorders: a retrospective study of patients seeking mental health services at a public psychiatric facility in Ghana

**DOI:** 10.1186/s13033-016-0095-1

**Published:** 2016-10-03

**Authors:** Abdallah Ibrahim, Sidua Hor, Ozge S. Bahar, Duah Dwomoh, Mary M. McKay, Reuben K. Esena, Irene A. Agyeponge

**Affiliations:** 1 School of Public Health, University of Ghana, P.O. Box LG13, Accra, Ghana; 2 Catholic Relief Services, RT 70 Gumani Residential Area, Tamale, Ghana; 3 Silver School of Social Work, New York University, New York, NY 10003 USA; 4 Ghana Health Service, Private Mail Bag, Ministries, Accra, Ghana; 5 The Brown School, Washington University in St. Louis, St. Louis, MO 63130 USA

**Keywords:** Pathways to care, Pantang Psychiatric Hospital, Mental disorders, Ghana

## Abstract

**Background:**

The process to seek for care by patients who experience episodes of mental disorders may determine how and where they receive the needed treatment. This study aimed to understand the pathways that people with mental disorders traversed for psychiatric services, particularly where these individuals will first seek treatment and the factors that influence such pathways to mental health care.

**Methods:**

A cross-sectional study conducted at Pantang psychiatric hospital in Accra, Ghana involving 107 patients of ages 18 and older and their family members. The study adapted the World Health Organization’s (WHO) pathway encounter form to collect information about patients’ pathway contacts for psychiatric care. Chi Square test was done to determine patients’ first point of contact and any association between the independent variables (clinical diagnosis and socio-demographic factors) and first pathway contact. Multiple regression analyses were also done to estimate the odds of patients’ first pathway contact.

**Results:**

Overall, nearly 48 % of patients initially contacted non-psychiatric treatment centers (faith-based, traditional healers and general medical practitioners) as their first point of contact for treatment of mental disorders. A little more than half of the patients went directly to the formal public psychiatric facility as their first point of contact for care of their mental disorders. Patients’ occupation was significantly associated with their first point of contact for psychiatric care (*χ*^2^ = 6.91; p < 0.033). Those with secondary education were less likely to initially seek care from the formal public psychiatric hospital compared to those with no formal education (uOR = 0.86; 95 % CI 0.18–4.08).

**Conclusion:**

Patients used different pathways to seek psychiatric care, namely direct pathway to a psychiatric hospital or through transition from informal non-psychiatric service providers. Since nearly half of patients do not initially seek mental health care directly at the formal psychiatric facility, it is important for the government of Ghana to increase funding to the mental health authorities in Ghana as a matter of priority so that more individuals can be identified and integrated into mainstream psychiatric treatment and general health facilities where there are trained Community Mental Health Officers (CMHO) and Clinical Psychiatric Officers (CPO) to provide early intervention and treatment.

## Background

The World Health Organization (WHO) has reported that approximately 2.2 million Ghanaians suffer from mental disorders, and 650,000 of that suffer from severe mental disorder [1]. It is also reported that there is a significant treatment gap (the number of people with mental disorders who are unable to get treatment) among those with mental disorders in Ghana, which is estimated to be at about 98 percent [1, 2].

In developing countries, including those in sub-Saharan Africa, many people with mental disorders prefer to consult traditional healers either as their first point of contact in the pathway to biomedical mental health services or as their sole mental health service providers [3]. Numerous factors influence patients’ pathways to seeking mental health care, and depending on the nature of the initial pathway, it can ultimately lead to delays in seeking treatment or avoiding formal treatment altogether for people with mental disorders, which consequently perpetuates the treatment gap in mental health care [4]. In most places, especially in the developing countries such as those in Africa, including Ghana, people with mental disorders tend to seek treatment from a wide variety of sources, including the formal and informal places, if they decide to seek mental health treatment at all [3].

In parts of Nigeria for instance, nearly 80 % of people with mental disorders initially sought mental health care at informal places of care including the priests, spiritualists, or herbalists [5]. Also, nearly 70 % of patients diagnosed with schizophrenia in Lagos, Nigeria, are known to have consulted spiritualists or traditional healers as their first point of contact in the pathways of seeking treatment for their mental disorders [6]. In South Africa, the Zulu people believe that Western medicine is very useful when treating physical illnesses, however, when it comes to mental illness, they believe Western medicine (or formal psychiatric care) could be ineffective because in their view, mental disorders can only be understood by the traditional healers from within their culture [7]. Hence, their pathway for mental health care may involve a delay in initially seeking care at the formal psychiatric facility.

Understanding the pathways to seeking mental health care can help explain delays in seeking professional mental health in an African environment such as Ghana. Delays in seeking early mental health care from a trained professional or at a facility equipped to manage psychiatric issues can result in poor clinical and functional outcome at a later point in time [8–10].

Factors that influence pathways to mental health care seeking in sub-Saharan African countries manifest at multi-levels, including at the individual, societal, organizational and at the health system level. In Uganda for instance, factors across different levels are at play at once. Fear of being diagnosed as suffering from mental illness, distrust towards the formal mental health system, and lack of confidence in health professionals make people hesitant to seek initial treatment directly at professional mental health providers [11]. Even the choice of where and when to first seek mental health care often depends on what is the individual’s mental disorder and/or his or her family believe is the cause of the disorder [11, 12]. Generally in Africa, mental illness is often believed to be caused by spiritual factors and for that matter some people with mental disorders would usually seek the services of African traditional healers first, before they consider seeking or exploring Western medical care [4].

In majority of less developed countries, the mental health system is often a low public health priority. Some studies have revealed that mental health care in many low and middle-income countries (LMIC), particularly those in Africa, including in Ghana, are consistently under-resourced [2, 3, 13]. In Ghana, there is relatively limited number of psychiatrists and public mental health facilities to provide the crucial early intervention for people experiencing mental disorders. Although there are a few regional psychiatric hospitals in three Regions of Ghana (Brong Ahafo, Ashanti and Volta Regions), the majority of the limited psychiatrists and all of the large public national mental health facilities are located mainly in only two southern regional cities of Ghana’s ten administrative regions [14]. As such many of those in need of mental disorder treatments in the other regions do not adequately receive the requisite psychiatric services at the large public mental health facilities at all. Instead, they prefer or are often left to seek care from the informal community resources such as the traditional and faith-based healers or at medical facilities with a varying quality of services and effectiveness [2, 15].

Even when system-level changes around mental health services are introduced, access to mental health services may remain limited, affecting the pathways to seeking mental health care. In 2012, the Parliament of Ghana passed the Mental Health Act (MHA-846) that aimed at: increased and improved access to mental health care; regulating mental health practices in private and public treatment centers; combating discrimination against people with mental disorders; and defining and limiting treatment for mentally ill patients without informed consent [16]. The new legislation essentially intended to completely overhaul and restructure Ghana’s public mental health system to change the way people sought mental health care. However, since its passage, some noticeable and commendable improvements have been made to the system but not the total and complete implementation of the key provisions of the law, especially in areas of improved access to mental health services in all communities where people live [2, 16].

Inadequate community-based formal mental health services therefore leaves the faith-based (or prayer camps) and traditional healers as the relatively ideal and viable avenue for mental health treatment. Despite reports of human rights abuses at the faith-based healing centers (e.g., being chained to trees, deprived of food and/or water), these avenues for treatment of mental disorders remain very popular in West Africa [17]. This is partially due to the belief that in African countries, including Ghana, mental illness is caused by evil spirits and the lack of formal mental health treating facilities [8, 18]. Consequently, patients who experience the onset of mental disorders seek help from the churches, prayer camps, imams (Islamic spiritual leaders) and other shrines wherever they are available across most part of Ghana.

Recent research findings show that in developing countries where severe psychotic disorders have been recorded, many of those in need of psychiatric treatment do not initially approach the formal psychiatric service providers at all, but seek the care of this informal community mental health service providers [3, 4, 11, 19]. Thus, some amount of time is lost before the initiation of treatment at the formal public mental health facilities, which results in poor response to psychiatric treatment and, consequently, lowers quality of life.

Generally, there is dearth of research in Ghana on psychiatric care, particularly on the pathways to care, yet knowledge of such pathways and the factors that influence them is crucial for public mental health service delivery in the country. The only known studies available looked at care seeking in areas where public psychiatric care is unavailable. Given the gap in the literature on pathways to psychiatric care in Ghana, this study sought to describe the distribution of first pathway contacts for psychiatric care by individuals with mental disorders attending the outpatient unit of a large public psychiatric facility. It also aimed at assessing possible link between socio-demographic factors and patients’ pathways before they sought care at the Pantang Psychiatric hospital in the Greater Accra region of Ghana.

## Methods

This cross-sectional study collected retrospective information from patients on their care seeking pathways that led them to the outpatient unit of the Pantang Psychiatric hospital. The study adapted the World Health Organization’s (WHO) collaborative ‘Pathway Study’ encounter form, which was designed for use in series of studies to understand care-seeking and treatment pathways of patients with mental disorders before they sought formal mental health care [20, 21].

### Study site

The study was conducted at the Pantang Psychiatric Hospital (Pantang), the largest of the three large public psychiatric hospitals in Ghana. The hospital is located in the Greater Accra region and started operation in 1975. The Pantang psychiatric hospital was originally built by Ghana’s first president to become a Pan African Mental Health Center for Research into Neuro- Psychiatric Conditions. It was also intended to decongest the main Accra Psychiatric Hospital. The Pantang psychiatric hospital was purposively selected for this study because it serves a large number of individuals with a variety of mental disorders and has other units that offer non-mental health services, including primary health care, and reproductive and child health services. Although psychiatric services at Pantang are supposedly free, patients are routinely asked to make a co-payment for things such as folders and medication, among others.

### Study population

The study interviewed adult patients to obtain their pathways information for mental health services. Adult significant others (family members, relatives or friends 18 years or older), who accompanied patients to the out-patient department of Pantang psychiatric hospital, were also present for the interviews. Where patients were unable to provide responses, their family members present were allowed to provide the responses.

### Study sample

The study questionnaires were administered to outpatient attendees at the psychiatric facility within a 2 week period in June 2015. The questionnaires were administered face-to-face to 107 study participants who were purposively selected to determine their pathways for psychiatric care. The sample size used was based on another study that compared treatment seeking behavior of mentally ill and sickle cell patients in Ghana [22]. Key variables such as religion, occupation, distance and marital status that are documented to influence patients’ decisions to seek mental health care at the formal public psychiatric hospital and informal psychiatric providers (e.g., traditional healers, religious prayer camps, and non-psychiatric general health centers) were used to determine patients pathways at the onset of their mental disorders [8, 23].

### Data collection tools and techniques

A semi-structured interviewer-administered questionnaire was developed for this study based on the pathway encounter form developed for the WHO collaborative study [21]. The tool was used to collect data on the number of patients with mental disorders that sought services at the various psychiatric service providers in both the formal and informal sectors in Ghana. This enabled us to do an estimated comparison of people with mental disorders that went to the traditional healers, religious (faith-based prayer camps) and non-psychiatric community/medical care providers before those individuals attended the outpatient unit of the Pantang public psychiatric hospital for mental health care services.

### Data analysis

The completed questionnaires were coded and entered into Microsoft Excel where the data was checked for errors and then exported to Stata SE Version 13.0 for analysis of the data (StataCorp, College Station, TX). Chi square and Fishers Exact test statistics were used, where appropriate, to estimate the places patients’ first sought psychiatric care against their socio-demographic characteristics as part of the pathway to psychiatric care. Since distance to psychiatric hospitals was not normally distributed, it was transformed to normality using inverse square root transformation and formally tested using the Shapiro-Francia normality test with the Box-Cox transformation. The mean distance from area of residence between those who first sought psychiatric care in a formal psychiatric hospital and all others who sought care at a non-public psychiatric place was compared using the standardized t-tests.

Univariable and multivariable binary logistic regression analysis were performed to assess the relative influence of socio-demographic characteristics on the first place patient sought psychiatric care (measured as ‘1’ if patients did not first seek psychiatric care in a public psychiatric hospital and ‘0’, for otherwise). One hundred bootstrap replications were used to estimate standard errors and confidence interval for all parameter estimates. Although most of the covariates studied were not statistically significant at 5 % level of significance, in building the nested model we included all variables with p-values less than 0.35 at multivariable stage. Akaike and Bayesian information criterions were used to compare nested models.

## Results

### Characteristics of study participants and mental health care seeking pattern

A total of 107 psychiatric patients participated in the study, comprising of 53 (49.5 %) males and 54 (50.5 %) females. The mean age was 37.4 (SD = 14.9) years with the youngest and the oldest person aged 18 years and 82 years respectively. In regards to level of education, 23.4 % had tertiary education and more than one-third (39.3 %) had basic education. More than half (57.9 %) of the total sampled respondents were single and 24.3 % were married. Seventy-five percent were Christian and 40.2 % were unemployed (Detailed description of the study participants can be found in Table 1). The most prevalent mental disorder among the study participants was Schizophrenia (33.3 %).

**Table 1:** Socio-demographic/economic characteristics of study participants

Item	Frequency	Percentage
*Sex*		
Male	53	49.5
Female	54	50.5
*Age (years)*		
<20	5	4.7
20–29	33	30.8
30–39	29	27.1
40–49	19	17.8
50–59	11	10.3
≥60	10	9.4
*Educational level*		
No/some formal education	14	13.1
Basic education^a^	42	39.3
Secondary education^b^	26	24.3
Tertiary	25	23.4
*Marital status*		
Single/never married	62	57.9
Married	26	24.3
Widowed	6	5.6
Divorced	8	7.5
Separated	5	4.7
*Distance to psychiatric hospital in km (median;iqr)*	107	(32.0; 63.5)^c^
*Religion*		
Christian	92	86
Islam	13	12.2
Others	2	1.8
*Occupation*		
Unemployed	43	40.2
Self-employed	44	41.1
Public servant	11	10.3
Retired	2	1.9
Other	7	6.5
*Ethnicity*		
Akan	40	37.4
Ewe	27	25.2

Distance to psychiatric hospital (in KM)

^a^Primary/Junior High

^b^Senior High/Vocational

^c^Represents median and inter-quartile range (iqr)

### Treatment and psychiatric care seeking

Of the 107 participants interviewed, the overall proportion of patients that first sought care at the psychiatric hospital was 52.3 %; another 23.3 % sought non-psychiatric treatment from religious or traditional healing centers as their first contact; 21.5 % sought treatment from non-psychiatric general hospital as first point of contact; and 2.9 % sought help from community health nurse and other community medical practitioners as their first point of mental health care contact before eventually coming to the Pantang psychiatric facility.

In relation to the duration of mental illness prior to the time of interview, 26 % has had a mental disorder for 11 or more years; 55 % had mental disorders for 1–10 years and approximately 19 % had mental disorders for less than a year. In regards to manifestation of symptoms of mental disorder and first pathway contact for mental disorder treatment, 77.6 % of the patients reported to have sought mental health service at the time their symptoms manifested from the first place they sought mental health care, either at the psychiatric or non-psychiatric facility.

Half of those diagnosed with Schizophrenia and substance use disorders initially sought psychiatric care from non-psychiatric treatment facility or centers before seeking care at the Pantang public psychiatric hospital. Most of the patients diagnosed with mental disorders such as psychotic disorder, depression and bipolar affective disorder first sought treatment from the psychiatric facility compared to those with other mental disorders. Table 2 shows the distribution of type of mental disorder and first pathway contact.

**Table 2:** Distribution of type of mental disorder and first pathway contact

Mental disorder (Diagnosis)	Religious/traditional healer	General medical service	Psychiatric service
	N (%)	N (%)	N (%)
Bipolar affective disorder	4 (44.4)	0 (0.0)	5 (55.6)
Depression	0 (0.0)	5 (38.5)	8 (61.8)
Mood disorder	1 (20.0)	2 (40.0)	2 (40.0)
Psychotic disorder	4 (14.8)	8 (29.6)	15 (55.6)
Schizophrenia	11 (30.6)	7 (19.4)	18 (50.0)
Seizure disorder	2 (33.3)	2 (33.3)	2 (33.0)
Substance abuse	3 (30.0)	2 (20.0)	5 (50.0)
Others	2 (22.2)	4 (44.4)	3 (33.3)

### Bivariate analysis of factors associated with place patients first sought psychiatric treatment

The proportion of females initially seeking care at non-psychiatric facility was slightly higher (51 %) compared to female who first sought care at formal psychiatric facility (49 %). The proportion of patients with Basic Education initially seeking care at the non-psychiatric facility (39.2 %) was almost the same as those who initially went to the psychiatric facility (39.3 %). Chi square test of independence results showed a statistically significant difference in first place of seeking psychiatric care among the different types of occupation (χ^2^ = 6.91; *df* = 2; p < 0.033). Self-employed patients were more likely to first seek care in non-psychiatric health facility compared to the unemployed and public servants. The single/never married group was also more likely to first seek care at non-psychiatric facility, but this was not statistically significant (Table 3).

**Table 3:** Cross-tabulation of socio-demographic characteristics and place patients first sought psychiatric treatment

Place patients first sought psychiatric treatment—n(%)				
Socio-demographic characteristics	Psychiatric hospital	Non-psychiatric hospital	*χ* ^2^ (df)	p value
*Sex*				
Male	28 (50.0)	28 (50.0)	0.01 (1)	0.919
Female	25 (49.0)	26 (51.0)		
*Age (years)*				
<20	3 (5.4)	2 (3.9)		
20–29	14 (25.0)	19 (37.3)		
30–39	16 (28.6)	13 (25.5)	Exact	0.734
40–49	12 (21.4)	7 (13.7)		
50–59	5 (8.9)	6 (11.8)		
≥60	6 (10.7)	4 (7.8)		
*Educational level*				
No formal education	7 (12.5)	7 (13.7)		
Basic education^a^	22 (39.3)	20 (39.2)	0.06 (3)	0.997
Secondary education^b^	14 (25.0)	12 (23.5)		
Tertiary	13 (23.2)	12 (23.5)		
*Marital status*				
Single/never married	28 (50.0)	34 (66.7)		
Married	16 (28.6)	10 (19.6)	Exact	0.344
Widowed	5 (8.9)	1 (2.0)		
Divorced	4 (7.1)	4 (7.8)		
Separated	3 (5.4)	2 (3.9)		
*Religion*				
Christian	46 (82.1)	46 (90.2)	1.44 (1)	0.231
Others	10 (17.9)	5 (9.8)		
*Occupation*				
Unemployed	28 (50.0)	15 (29.4)		
Self-employed	22 (39.2)	22 (43.1)	6.91 (2)	0.033*
Public servants	6 (10.7)	14 (27.5)		
Distance (in km) (mean ± SD)^c^	0.17 ± 0.07	0.16 ± 0.06	1.35 (105)^d^	0.1773
*Ethnicity*				
Akan	20 (35.7)	20 (39.2)	0.14 (1)	0.708
others	36 (64.3)	31 (60.8)		

*χ* ^*2*^ Chi square test statistic; *df* degrees of freedom; numbers in parentheses indicate column percentages; *Exact* estimates from Fishers Exact test

* p < 0 0.05

^a^Primary/junior high

^b^Senior high/vocational

^c^ $$\left( {\frac{1}{{\sqrt {\text{distance}} }}} \right)$$ transformation

^d^Estimates the studentized t-test statistic

### Multivariable analysis of factors associated with first pathway contact for psychiatric care

Table 4 shows the results of multivariable analysis of factors associated with why patients did not first seek mental treatment at the Pantang psychiatric hospital. With individuals under 20 years as the reference group, those aged 20 to 39 were more likely to seek mental health care at a non-psychiatric facility as a preferred first point of contact, which was not statistically significant. Although most of the variables were also not statistically significant at the univariable stage of the analysis, they were included in the multivariable analysis due to previous relationship with the outcome variable from existing literature. This resulted in a two different nested statistical model that were fitted based on a cut-off point of p < 0.35 and the model performance for the two models were tested based on BIC (model 2 had the minimum BIC and therefore chosen as our final model) (Table 5). The odds of first seeking psychiatric care in a non-psychiatric health facility is almost two times higher for the self-employed and 4.4 times higher among public servants compared to those who are unemployed (95 % CI 1.2–16.3, p < 0.011); (Table 5).

**Table 4:** Univariate and multivariate analysis of factors associated with a non-public psychiatric health facility as first place patients sought care prior to Pantang hospital

Socio-demographic characteristics	uOR	95 % CI	Unadjusted p value	aOR	95 % CI	Adjusted p value
*Sex*						
Male	ref			ref		
Female	1.04	0.44–2.45	0.928	0.77	0.21–2.79	0.676
*Age (years)*						
<20	ref			ref		
20–29	2.04	0.37–11.25		3.95	0.17–93.94	
30–39	1.22	0.20–7.36		2.15	0.07–62.07	
40–49	0.89	0.13–6.03		2.09	0.03–148.30	0.602
50–59	1.8	0.26–12.55		3.58	0.10–133.66	
≥60	1	0.14–7.34	0.382	1.74	0.01–212.47	
*Educational level*						
No formal education	ref			ref		
Basic education^a^	0.9	0.19–4.33		0.35	0.01–10.27	
Secondary education^b^	0.86	0.18–4.08		0.21	0.01–7.48	0.264
Tertiary	0.92	0.15–5.83	0.916	0.11	0.00–6.02	
*Marital status*						
Single/never married	ref			ref		
Married	0.51	0.22–1.22		0.25	0.04–1.68	
Widowed	0.16	0.04–0.69		0.05	0.01–0.80	
Divorced	0.82	0.09–7.58		0.46	0.02–9.74	0.172
Separated	0.54	0.08–3.59	0.254	0.28	0.01–5.77	
*Religion*						
Christian	ref			ref		
Others	0.5	0.12–2.01	0.194	0.31	0.03–3.12	0.301
*Occupation*						
Unemployed	ref			ref		
Self-employed	1.87	0.76–4.59		2.61	0.48–14.24	0.007*
Public servant	4.36	1.07–17.70	0.011*	13.65	1.11–16.40	
*Distance in km*	0.99	0.99–1.00	0.860	0.99	0.98–1.00	0.794
*Ethnicity*						
Akan	ref			ref		
others	0.86	0.41–1.81		0.81	0.21–3.10	0.961

uOR and aOR are unadjusted and adjusted odds ratio respectively based on binary logistic regression, ref represents the reference category of the variable

Standard error and confidence interval estimation were based on 100 bootstrap replications

* p < 0.05

^a^Primary/Junior High

^b^Senior High/Vocational

**Table 5:** Nested model building based on the Akaike Information Criterion and Bayesian Information Criterion on seeking care in psychiatric hospital

Variables	Model 1	p value	Model 2	p value
	aOR (95 % CI)		aOR (95 % CI)	
*Education level*				
No formal education	ref		**	**
Basic education^a^	0.40 (0.07–2.20)^c^			
Secondary education^b^	0.34 (0.05–2.23)	0.149		
Tertiary	0.16 (0.03–0.90)			
*Marital status*				
Single/never married	ref			
Married	0.22 (0.05–0.88)^c^		**	**
Widowed	0.05 (0.00–0.65)			
Divorced	0.38 (0.07–1.91)			
Separated	0.2 (0.07–1.91)	0.143		
*Religion*				
Christian	ref			
Others	0.3 (0.05–2.50)^c^	0.218	**	**
*Occupation*				
Unemployed	ref		ref	
Self-employed	2.99 (0.85–10.54)^c^		1.89 (0.66–5.32)	
Public servant	11.07 (1.21–101.28)	0.008*	4.36 (1.16–16.30)	0.011*
*Measures of Fit for logistic*				
AIC	1.357		1.356	
BIC	−341.424		−349.526	

*AIC* akaike information criterion, *BIC* Bayesian information criterion

* p < 0.05; ** Variables that were not used in the model

^a^ Primary/junior high

^b^Senior high/vocational

^c^ Variables used in the model

## Discussion

Overall, findings from this cross-sectional study provide some descriptive insight into the pathways that some people with mental disorders in Ghana take in their quest for psychiatric care. The findings reveal that nearly half of people with mental disorders who went to the public psychiatric facility had initially visited the non-psychiatric treatment centers as their first point of contact prior to visiting the formal public mental health service center at Pantang in the Greater Accra Region of Ghana. Although nearly half had initially been to other non-psychiatric providers, the findings show that the pathways of a little more than half of those with mental disorders initially took them directly to the formal psychiatric facility for treatment, which is similar to other studies in Ghana [22, 24].

Even though less than half of the patients in this study had initially sought care from non-psychiatric providers such as faith-based religious or traditional healers, similar observations were made in Ethiopia where religious healers and herbalists were first point of contact for some patients before they eventually went to the public psychiatric hospital [8]. This study’s finding of more than half of the patients going directly to the public psychiatric as their first pathway point of contact for mental health services seem consistent with similar finding in Nigeria where majority of patients sought treatment at a formal psychiatric care facility [12]. This study’s finding also raises a very important issue about access and affordability of mental health care services in Ghana. Even though mental health patients are generally not charged for mental health services at the public psychiatric facilities in Ghana, they routinely have to make out of pocket payments to access some mental health services at the Pantang psychiatric hospital, which has a relatively good facility for mental health care compared to others. The routine out of pocket expenses for mental health services can be a deterrent for some people who may not be able to afford it, which could result in those people preferring to initially visit non-psychiatric service providers as their first pathway contact until they are able to afford money to go to the large public psychiatric facility, especially at Pantang. However, if the government increase funding for psychiatric services as well as expand the national health insurance scheme (NHIS) to cover mental health services, it could enable increased access to care and also help eliminate some of the out-of-pocket costs that usually becomes a deterrent for individuals or families seeking formal psychiatric care.

The finding in this study of a majority of patients seeking care at the large psychiatric facility also point to a positive trend towards acceptance of the efficacy of formal psychiatric treatment at large public mental health facilities such as the Pantang psychiatric hospital in Ghana and in other places as an ideal place for initial contact for psychiatric care, especially since some Africans generally do not trust the formal or Western psychiatric services [7, 11, 25]. This is particularly important in Ghana and elsewhere in Africa where there is widespread misconception about mental disorders in general and in seeking mental health care from large psychiatric facilities in particular as part of pathways to care.

Contrary to expectations, the number of patients who initially made contacts with the non-psychiatric religious, traditional healers and the general medical practitioners was lower compared to those who made first contacts with the formal psychiatric hospital. The observation in this study of some patients initially seeking psychiatric help from the general medical practitioners was similar to observations made in Japan where referral to formal psychiatric facilities relied on the general medical sources [26]. However, most of the patients who visited non-psychiatric care centers as their first point of contact had eventually transitioned to the public psychiatric facility as their second point of contact for psychiatric service, presumably since patients’ visit to the general medical practitioners and specifically to religious and traditional healing centers may not have yielded the desired treatment outcomes. This observation is consistent with other findings in places such as England London where patients who made first contact with non-psychiatric centers, including general medical facilities would eventually make contact with the public psychiatric service providers as they transition from place to place in their pathway contacts for psychiatric treatment [27].

The majority of those with schizophrenia, psychotic disorders, depression and bipolar affective disorders in this study sought care at the Pantang public psychiatric facility as their first pathway contact compared to the other mental disorders that are among the top ten mental disorders in Ghana [28]. This finding among those diagnosed with schizophrenia in particular and other psychiatric conditions in general is consistent with other studies in Nigeria as well as in other reports regarding the type of mental disorders that usually lead to seeking institutional treatment in Ghana [6, 12, 13]. There is no specific explanation in this study as to why those diagnosed with schizophrenia for instance preferred the public psychiatric facility as their first point of contact other than the fact that schizophrenia patients constituted more than one-third of the total patients interviewed. Similarly, those with psychotic disorders who were in the majority in this study initially sought mental health care from the public psychiatric care facility, which is contrary to a study finding in Sudan where the majority of psychotic disorder patients sought treatment from traditional healing centers [29].

In this study, there was a significant association between employment status and pathway to care, especially among public servants who went to the non-psychiatric facility as the first point of contact. The observation about the link between employment and pathway contact is in line with another study in Ghana about use of traditional healers and modern medicine [30].

Generally, patients and their families would usually consider a number of pathways in search of treatment for mental disorders. Their decision is usually influenced by a number of factors, including socioeconomic status such as education and employment. Given the strong association between those with employment and non-psychiatric facility as the first pathway to care in this study, it suggests that the gainfully employed may not want the perceived stigma associated with being seen at a public psychiatric hospital and therefore chose to access care at the non-psychiatric service providers as their first point of contact. Therefore, it will be helpful to address the issues of stigmatization of mental health patients and their access to the needed healthcare facilities through public education. This is particularly important for Ghana and elsewhere in Africa where people with mental disorders are consistently stigmatized, especially among those with the severe form of mental disorders without access to treatment that roam the streets in Ghana and are occasionally harassed or even physically assaulted. Although addressing the stigma of mental illness was included in the provisions of the recent mental health law in Ghana, limited and slow implementation of the provision for public education to de-stigmatize mental disorders may provide a window of explanation as to why those who are gainfully employed or educated avoid seeking care from the public psychiatric treatment facilities as a first point of contact at the early onset of their mental disorders.

## Conclusion

Different pathways to seek psychiatric care have been described in this study. These include seeking: a direct pathway to a public psychiatric hospital at the onset of a mental health condition; direct pathway to a non-psychiatric general medical practitioner; and direct pathway to the faith-based or traditional healing centers. Even though the latter points of contact (religious prayer camps, traditional healers or general hospital) is a preference for some individuals as the initial place to seek mental health treatment, the presence of those patients at the large public psychiatric facility indicates that they eventually decided to transition to the more formal psychiatric treatment facility for mental health services in Ghana.

Since a little less than half of the patients in this study did not initially seek mental health care directly at the public psychiatric facility, it is important for the public mental health authorities in Ghana to recognize that it is important to increase funding to incorporate these faith-based and traditional healers as well as expand psychiatric care at the general medical health facilities as part of the mental health continuum of care. Doing so will enable people with mental disorders to access mental health treatment regardless of which door they initially choose to access mental health care. Hence, there would be no wrong door for mental health service seeking in Ghana. In fact, since commendable efforts are being made to improve the system following the passage of the new mental health law, we highly recommend that the government of Ghana should expand coverage of the national health insurance scheme to cover psychiatric patients at public psychiatric facilities. The Parliament of Ghana should also seriously consider approving an allocation from the national consolidated fund to shore up the mental health fund established to increase mental health care access nationwide.

### Study limitations

Although this is the first known retrospective study to determine and describe mental health care seeking pathway among outpatient mental health patients at a major public psychiatric facility in Ghana, it is not without some limitations. First, since the study included some family members as part of the respondents, it is possible that the information obtained may not be entirely accurate, especially since some family members may not have had all the facts or treatment history, especially among the patients with chronic mental disorders who have been in treatment for several years.

The retrospective nature of the study may have resulted in some inaccuracies with recall bias. Some of the patients in the study indicated to have had their first episode of mental disorders in more than 10 years. With the length of time since the patients first had their onset of mental disorders and the time of interviews, some family members or care givers of the study patients might have changed over time and therefore, the information that some family members provided about the patients’ pathways at the time of interviews could be inaccurate or very limited.

Additionally, majority of the participants lived in and around the Greater Accra Region even though they may have migrated from different parts of the country and so their proximity to the Pantang psychiatric hospital leveraged their geographical access to the facility compared to others in distant towns and villages. Furthermore, the ethnic distribution of the participants highlights the important role of geographical access to healthcare. The predominant ethnic groups in the study were Akans, Ewes, Ga-Adangbe and Krobos, which are all ethnic groups mostly found in the southern part of Ghana. Very few ethnic groups from the northern part of Ghana were recorded in this study. Therefore, this does not in any way suggest that the predominant ethnic groups in this study are more predisposed to developing mental health conditions or seeking care at one place or another. It only points to the possibility of their proximity to the facility.
