# Erratum to: Pathways to psychiatric care for mental disorders: a retrospective study of patients seeking mental health services at a public psychiatric facility in Ghana

**DOI:** 10.1186/s13033-016-0103-5

**Published:** 2016-10-18

**Authors:** Abdallah Ibrahim, Sidua Hor, Ozge S. Bahar, Duah Dwomoh, Mary M. McKay, Reuben K. Esena, Irene A. Agyepong

**Affiliations:** 1 School of Public Health, University of Ghana, P.O. Box LG13, Accra, Ghana; 2 Catholic Relief Services, RT 70 Gumani Residential Area, Tamale, Ghana; 3 Silver School of Social Work, New York University, New York, NY 10003 USA; 4 Ghana Health Service, Private Mail Bag, Ministries, Accra, Ghana; 5 The Brown School, Washington University in St. Louis, St. Louis, MO 63130 USA

## Erratum to: Int J Ment Health Syst (2016) 10:63 DOI 10.1186/s13033-016-0095-1

In the original publication of this article [1], one of the co-authors’ name was written incorrectly. ‘Irene A. Agyeponge’ should have been listed as ‘Irene A. Agyepong’. This change has been reflected in the original publication as well.
